# Colorectal cancers with microsatellite instability display mRNA expression signatures characteristic of increased immunogenicity

**DOI:** 10.1186/1476-4598-3-21

**Published:** 2004-08-06

**Authors:** Ayan Banerjea, Shafi Ahmed, Rebecca E Hands, Fei Huang, Xia Han, Peter M Shaw, Roger Feakins, Stephen A Bustin, Sina Dorudi

**Affiliations:** 1 Centre for Academic Surgery, Barts and The London Queen Mary School of Medicine and Dentistry. The Royal London Hospital, Whitechapel, London, E1 1BB, UK; 2 Department of Pharmacogenomics, Bristol Myers Squibb, 311 Pennington-Rocky Hill Road, Pennington, NJ 08534, USA; 3 Institute of Pathology, The Royal London Hospital, Whitechapel, London, E1 1BB, UK

## Abstract

**Background:**

Colorectal cancers displaying high-degree microsatellite instability (MSI-H) have an improved prognosis compared to microsatellite stable (MSS) cancers. The observation of pronounced lymphocytic infiltrates suggests that MSI-H cancers are inherently more immunogenic. We aimed to compare the gene expression profiles of MSI-H and MSS cancers to provide evidence for an activated immune response in the former.

**Results:**

We analysed tissue from 133 colorectal cancer patients with full consent and Local Ethics Committee approval. Genomic DNA was analysed for microsatellite instability in BAT-26. High-quality RNA was used for microarray analysis on the Affymetrix^® ^HG-U133A chip. Data was analysed on GeneSpring software version 6.0. Confirmatory real-time RT-PCR was performed on 28 MSI-H and 26 MSS cancers. A comparison of 29 MSI-H and 104 MSS cancers identified 2070 genes that were differentially expressed between the two groups [P < 0.005]. Significantly, many key immunomodulatory genes were up-regulated in MSI-H cancers. These included antigen chaperone molecules (HSP-70, HSP-110, Calreticulin, gp96), pro-inflammatory cytokines (Interleukin (IL)-18, IL-15, IL-8, IL-24, IL-7) and cytotoxic mediators (Granulysin, Granzyme A). Quantitative RT-PCR confirmed up-regulation of HSP-70 [P = 0.016], HSP-110 [P = 0.002], IL-18 [P = 0.004], IL-8 [0.002] and Granulysin [P < 0.0001].

**Conclusions:**

The upregulation of a large number of genes implicated in immune response supports the theory that MSI-H cancers are immunogenic. The novel observation of Heat Shock Protein up-regulation in MSI-H cancer is highly significant in light of the recognised roles of these proteins in innate and antigen-specific immunogenicity. Increased mRNA levels of pro-inflammatory cytokines and cytotoxic mediators also indicate an activated anti-tumour immune response.

## Background

Colorectal cancer remains a leading cause of cancer death in the Western world despite recent advances in surgery, radiotherapy and chemotherapy [1]. Immunotherapy has attracted attention as a novel treatment modality that may exploit the host immune response against tumour cells. However, definitive evidence that colorectal cancer cells can stimulate a specific immune response has been elusive.

Approximately 15–20% of sporadic colorectal cancers and nearly all large bowel malignancies in the Hereditary Non-Polyposis Colorectal Cancer (HNPCC) syndrome are characterised by widespread microsatellite instability [2,3]. Microsatellites are very short repetitive nucleotide sequences, distributed throughout the human genome, that are prone to insertion and deletion mutations during DNA replication. These mutations are normally corrected by the inherent proofreading capacity of DNA polymerase and a group of genes involved in mismatch repair (MMR). Defective mismatch repair allows the accumulation of errors in microsatellites and this is termed microsatellite instability (MSI). In HNPCC a germline mutation in a mismatch repair gene is inherited and a subsequent "second hit" leads to failure of MMR, resulting in MSI. In sporadic cancers epigenetic silencing by hypermethylation of the MMR genes has been implicated. Despite these differences in their molecular genesis the two groups share common clinicopathological features [4]. Several studies have confirmed that patients with tumours displaying a high degree of microsatellite instability (MSI-H) appear to possess a survival advantage over those with cancers that are microsatellite stable (MSS) [5-7]. This improvement in outcome appears to be an inherent feature of the unstable phenotype.

It has been shown that MSI-H cancers generate abnormal peptides that can be used to excite cytotoxic T cell responses in *in vitro *experiments [8,9]. These peptides may act as Tumour specific antigens (TSA's) *in vivo *and hence, excite a host immune response. In keeping with this observation MSI-H cancer is characterised by the presence of a significant infiltrate of lymphocytes, a feature that has been previously associated with better patient prognosis [10]. Lymphocytes that infiltrate tumour epithelium (intra-epithelial lymphocytes, IEL's) are specifically associated with improved survival and may be involved in an immune response [11]. Immunohistochemical analyses have shown that the IEL's infiltrating MSI-H colorectal cancers are predominantly cytotoxic, activated and release mediators of target cell death [12]. Follow-up analyses confirm improved survival in patients with these tumours [13]. Increased apoptosis has also been demonstrated in MSI-H cancers but the link between increased lymphocyte infiltrate and apoptotic cell death has not yet been proven. Some argue that these infiltrates are secondary phenomena with no biological relevance [14] and it has been suggested that intra-epithelial lymphocyte populations in MSI-H colorectal cancers simply represent proliferation of resident lamina propria lymphocytes with no immunological activation or role.

The development of high-density data analysis techniques such as microarray technology allows rapid gene expression profiling of tissue-derived RNA to give an mRNA expression signature for the tissue under study. The gene expression signature of a tumour microenvironment reflects the interactions between tumour, stroma and host response therein. We aimed to compare these signatures between groups of MSI-H and MSS colorectal cancers to identify genes that are differentially expressed between the two phenotypes. Specifically, we focus on genes involved in anti-tumour immune responses whose activity may be modified in colorectal cancers, in order to clarify the nature of any immune response in MSI-H colorectal cancer.

## Results

We analysed 133 colorectal tumours of which 29 (22%) tumours were identified as MSI-H (Table 1). This is at the upper end of accepted figures but reflects the frequency of MSI-H in a subset of our tumour bank that yielded high-quality RNA. The overall prevalence of MSI-H colorectal cancer in our tumour bank is lower (16%) and consistent with other large series. As expected the MSI-H group showed a statistically significant association with the right side of the colon (P < 0.0001, χ^2 ^test). During histological assessment each tumour was graded for lymphocytic infiltration on standard Haematoxylin and Eosin stained sections by a consultant pathologist (RF). Tumours with minimal or mild infiltration were scored 1, those with moderate infiltration scored 2 and those with pronounced lymphocytic infiltrates were scored 3. As expected the MSI-H cancer group had higher proportions of tumours with moderate and pronounced infiltration but this difference did not reach statistical significance (P= 0.287, χ^2 ^test for trend).

**Table 1:** Summary of patient demographics for microarray and RT-PCR analyses.

	**Microsatellite Stable (MSS)**	**Microsatellite Unstable (MSI-H)**
**Microarray analysis**		
Patients	104	27
Mean age (SD) (yrs)	69.6 (12.3)	65.5 (15.6)
Male: Female (%)	62:42 (60:40)	13:14 (48:52)
Cancers (n)	104	29
Right: Left (%)	28:76 (27:73)	19:10 (66:34)
Dukes' Stage A (%)	14 (13.5)	3 (10.3)
B (%)	48 (46.1)	15 (51.7)
C (%)	39 (37.5)	10 (34.5)
D (%)	3 (2.9)	1 (3.4)
Lymphocyte score 1(%)	78(75)	19(65.5)
2(%)	17(16.3)	6(20.7)
3(%)	9(8.7)	4(13.8)
**RT-PCR analysis**		
Patients	26	26
Mean age (SD) (yrs)	71.4 (13.6)	66.4 (15.1)
Male: Female (%)	14:12 (54:46)	12:14 (46:54)
Cancers (n)	26	28
Right: Left (%)	17:9 (65:35)	19:9 (68:32)
Dukes' Stage A (%)	2 (7.7)	3 (10.7)
B (%)	15 (57.7)	15 (53.6)
C (%)	8 (30.8)	10 (35.7)
D (%)	1 (3.8)	0

For RT-PCR analysis groups were matched for age, tumour side and Dukes' Stage. Two MSI-H patients had two tumours each.

An initial comparison of the gene expression profiles of MSI-H versus MSS tumours, using a parametric unpaired t-test (with Welch's correction for unequal variances) and the Benjamini and Hochberg False Discovery Rate (multiple correction method), identified 2070 genes that were differentially expressed at a significance of p < 0.005 (Additional Table 1 ). This represents 9.3% of those included on the chip and statistically less than 0.5% of these genes would be selected by chance. 1293 genes (62.5%) had significantly increased signal intensity in MSI-H cancers and 777 genes (37.5%) had reduced signal intensity.

**Figure 1:**
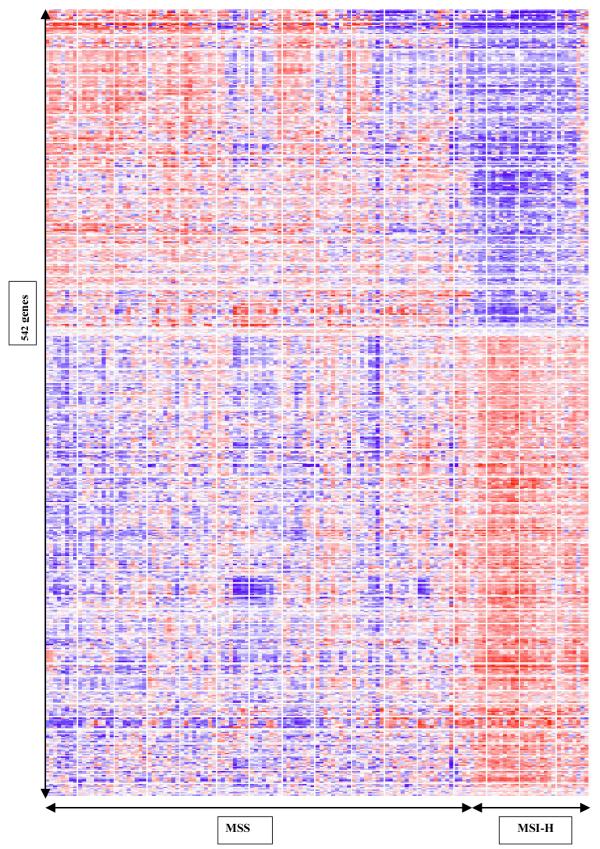
Two-way hierarchical clustering by the 542 most significantly differentially expressed genes between MSS and MSI-H colorectal cancers. Samples arranged along the x-axis and genes along the y-axis. Each square represents the expression level of a given gene in an individual sample. Red represents increased expression and blue represents decreased expression relative to the normalised expression of the gene across all samples. Samples with similar gene expression profiles are clustered together.

The clear differences between the two groups can be demonstrated in a cluster map (Figure 1). This was generated using 542 of the most significant genes in our list, selected by performing a group comparison at p < 0.05 and the more stringent Bonferoni multiple testing correction (Additional Table 1 ). The expression signatures of the MSI-H group on the right of the cluster map display marked homogeneity, in contrast to the heterogeneous MSS cancers. Four tumour profiles came from patients who satisfied family history criteria for HNPCC (Amsterdam criteria). These profiles were clustered amongst the other MSI-H cancers but did not form a distinct group. Analysis after exclusion of these HNPCC tumour profiles yielded very similar gene lists (Additional Table 2 ). The small number of HNPCC MSI-H profiles (<5) precluded meaningful comparison of expression profiles with the sporadic group.

1328 of our 2070 genes (64.2%) had a recognised function. We noted differences in several cancer-related genes that were consistent with our existing knowledge of the genetic profiles of MSI-H colorectal cancers (Table 2). Notably, the mismatch repair gene hMLH1 had reduced signal intensity in our MSI-H group, as did the TGFβ RII and IGFIIR genes. The mismatch repair gene PMS2 was also underexpressed in our MSI-H cancers (P = 0.003, Fold change 1.4). The mRNA of TP53 gene was more abundant in MSI-H tumours when compared to MSS tumours. Similarly, the β catenin gene also had a high signal in our MSI-H tumours. Significantly, several transcripts related to the heat shock protein family (HSP 70, 110 and 90) were up-regulated in MSI-H tumours (Table 2), as were several other genes that may be involved in a putative antigen-directed immune response. We focussed on genes relevant to immunological responses but many other interesting differences are evident in our list but are not discussed in this paper.

**Table 2:** A highly selective list of gene specific probes that are differentially expressed (P < 0.005) between MSI-H and MSS colorectal cancers with a fold-change of at least 1.5.

**Genes Up-regulated in MSI-H Colorectal Cancer**		
**Gene name**	**P value**	**Fold change**

Catenin (cadherin assoc. protein) beta 1	6.5 × 10^-12^	2.9
Interleukin 8*	1.7 × 10^-4^	2.8
Granulysin*	1.3 × 10^-7^	2.8
Caspase 2*	6.0 × 10^-8^	2.4
Interleukin 24	0.004	2.3
Heat shock protein (HSP 110 family)*	2.4 × 10^-5^	2.2
TP53 (Li Fraumeni)*	2.5 × 10^-4^	2.2
Toll-like receptor 2 (TLR-2)	1.9 × 10^-4^	1.9
Heat shock protein 70*	3.5 × 10^-6^	1.8
Granzyme A	0.001	1.7
Interleukin 1β	0.004	1.7
Survivin	0.001	1.6
Calreticulin	2.9 × 10^-5^	1.6
Human Natural killer Cell enhancing factor	0.001	1.6
CD68 antigen	4.8 × 10^-4^	1.6
ICAM 1 (CD54)	0.003	1.6
Interleukin 18 (interferon-gamma inducing factor)*	0.003	1.5
Interleukin 7	4.6 × 10^-4^	1.5
Interleukin 15*	0.002	1.5

**Genes down-regulated in MSI-H colorectal cancer**		

**Gene name**	**P value**	**Fold change**

Insulin-like growth factor 2 (somatomedin A)	1.9 × 10^-5^	4.3
TGFβ RII	9.3 × 10^-5^	3.0
hMLH 1*	6.7 × 10^-5^	2.4

P values denote results of Welch's t test with Benjamini and Hochberg False Discovery Rate. * denotes genes selected for RT-PCR analysis.

**Figure 2:**
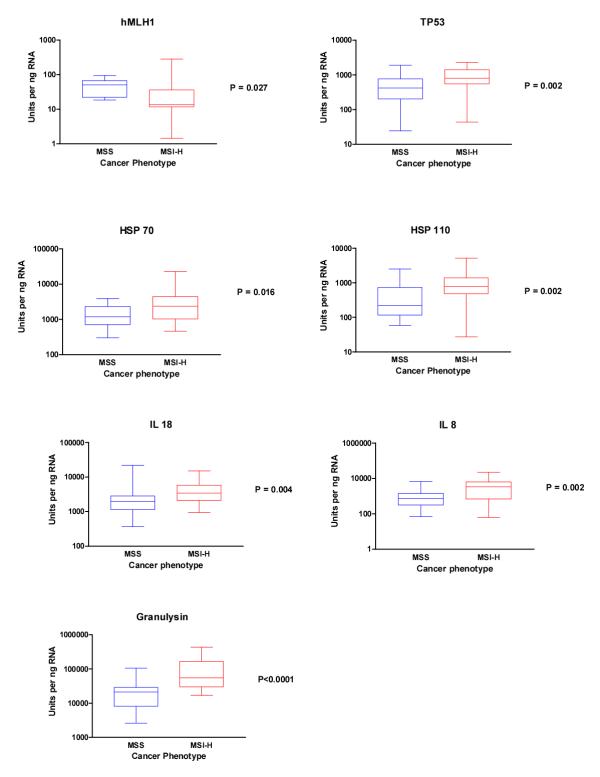
Boxplots showing RT-PCR data analysis of seven genes of interest:A hMLH1, B TP53, C HSP-70, D HSP-110, E IL-18, F IL-8, and G Granulysin. Data analysed using non-parametric Mann Whitney test (P values as shown).

To validate our microarray results we used real time RT-PCR to confirm the findings on nine genes of immunological interest. The RT-PCR results confirmed the significant differences between the two groups (Table 1) in seven out of the nine genes selected. The mismatch repair gene hMLH1 was significantly down-regulated in MSI-H (Figure 2a), whilst transcription of TP53 was significantly higher in the unstable group (Figure 2b). Similarly the heat shock protein (HSP) 70 (Figure 2c) and HSP-110 (Figure 2d), the Interleukins (IL) 18 (Figure 2e) and IL-8 (Figure 2f), and the protease Granulysin (Figure 2g), were all significantly up-regulated in MSI-H when compared to the MSS group. Two analyses, IL-15 (p = 0.17) and Caspase 2 (p = 0.16), had reduced sample numbers in each group and did not reach statistical significance. However, both showed trends of up-regulation in MSI-H cancers consistent with the microarray analysis.

## Discussion

Our study examines the differences in overall gene expression profiles in the tumour micro-environments of MSI-H and MSS colorectal cancers. The observation that a large number of pro-inflammatory genes are upregulated in MSI-H colorectal cancer is a strong indicator that an immune response is indeed activated in these tumours. These results support the notion that the lymphocytic infiltrates in these cancers represent immune activation rather than simple proliferation of resident lymphocytes.

The exact nature of the immune response remains unclear but our novel observation that heat shock proteins are upregulated in MSI-H colorectal cancer may be highly significant. Microarray data analysis demonstrated that several members of the HSP family are up-regulated in MSI-H cancers (Table 2) and RT-PCR analyses confirmed increased levels of both HSP-110 and HSP-70 mRNA in our MSI-H cancers. Heat shock proteins have roles in both innate and adoptive immunity and have excited much interest as natural adjuvants for immunotherapy [15,16]. Some members of the HSP family act directly to excite an innate immune response that might be more marked in MSI-H colorectal cancer. Such a response might be mediated by Natural Killer cells that characteristically release Granulysin to induce tumour cell death, a recognised feature of MSI-H colorectal cancer, which in turn releases intra-cellular TSA's. Alternatively, the concept of "effete malignancy", in which accumulation of mismatch errors overwhelms the tumour cells' viability, [17] may explain increased tumour cell death and release of TSA's. In fact, these two possible explanations of increased apoptosis in MSI-H colorectal cancers may actually be complementary rather than mutually exclusive. However, the release of TSA's into the tumour micro-environment is the pivotal step in the generation of an antigen-specific immune response.

Heat shock proteins act as chaperone proteins in the processing and presentation of antigenic peptides [15,16]. They promote antigen uptake and induce expression of antigen presenting and co-stimulatory molecules on dendritic cells. By example, HSP-70 has been shown to recruit dendritic cells (and T cells) and enhances their ability to uptake antigen [18]. This is a crucial step in the cross-priming of dendritic cells necessary for an antigen-directed immune response [19]. The CD68 antigen, expressed by immature dendritic cells and macrophages that are ready to take up antigen, is included in our list of genes up-regulated in MSI-H colorectal cancer, as is the TLR-2 gene, one of a family of receptors to which HSP's bind to activate dendritic cells.

Heat Shock Proteins have also been shown to induce cytokine profiles that promote antigen-specific responses. This role may underscore our observation that several immunogenic interleukins are up-regulated in MSI-H cancers. An important function of these cytokines is to promote the presentation of antigen by dendritic cells (and macrophages) to effector T cells. This represents another crucial step in the development of an antigen-specific immune response, prior to the interaction of primed cytotoxic (CD8+) and helper (CD4+) T cells with the tumour cells. This interaction subsequently results in lytic tumour cell death. Specifically, such cytokines also modulate which arm of the T helper system is activated: Th1 activity favours immune activity whilst Th2 pathways favour tolerance. In this context, our data reveals significant up-regulation of IL-18 and other pro-inflammatory cytokines in MSI-H cancers that promote Th1 activity.

Our microarray data are validated by the existing knowledge of key gene expression in MSI-H colorectal cancers. The presence of hMLH1 mRNA was reduced in MSI-H cancers, as shown by both microarray and RT-PCR analyses. This observation reflects the fact that hMLH1 is frequently silenced, due to promoter region hypermethylation, in sporadic cancers, which formed the majority of our MSI-H group. Other genes known to be affected by the MSI pathway such as TGFβ RII, IGFIIR, TP53, APC, β catenin and Bcl-2 were also differentially expressed between our two groups [20,21]. The inclusion of HNPCC cancers within our MSI-H group appears not to affect the differentially expressed genes we identify and this is likely to reflect their small number. Accrual of further HNPCC gene expression profiles should allow us to sub-analyse the MSI-H group in the future. However, the immunological focus of this paper appears unaffected by any differences in the biology of these cancers.

As expected a large number of pro-apoptotic genes were upregulated in our MSI-H group as these cancers are characterised by increased apoptosis (Table 3). The increased levels of Bcl-2 related transcripts are consistent with previous findings despite the anti-apoptotic functions of this gene [22,23]. Clearly, the interaction between pro-and anti-apoptotic agents in MSI-H cancers is complex and needs further elucidation.

**Table 3:** Additional genes related to apoptosis and the Major Histocompatibility Complex shown to be up-regulated in MSI-H colorectal cancer in comparison to MSS cancers.

**Apoptosis related genes up-regulated in MSI-H colorectal cancers (P < 0.005, Benjamini and Hochberg False Discovery Rate)**	
**Gene name**	**Fold change**

TNF-induced protein	1.9
Tumour necrosis factor receptor superfamily 10d	1.9
Tumour necrosis factor receptor superfamily 9	1.7
Tumour necrosis factor receptor superfamily 10b	1.7
Tumour necrosis factor receptor superfamily 6	1.6
Death-associated protein kinase 1	1.5
DNA fragmentation factor, beta polypeptide (caspase-activated DNase)	1.4
Bcl-2-related protein A1	2.2
Bcl-2/adenovirus E1B 19 kDa interacting protein 3-like	1.8
Bcl-2 antagonist/killer	1.4
Bcl-2 associated athanogene 3	1.4
Bcl-2/adenovirus E1B 19 kDa interacting protein 1	1.2
Baculoviral IAP repeat-containing 3	1.6
Baculoviral IAP repeat-containing 2	1.2
Chemokine (C-X-C motif) receptor 4	1.8
CD27-binding (Siva) protein	1.5
Thioredoxin-like, 32 kDa	1.4
Mitogen-activated protein kinase kinase kinase 5	1.4
Microtubule associated protein tau	1.4
Macrophage erythroblast attacher	1.3
Testis enhanced gene transcript (BAX inhibitor 1)	1.2
**Major histocompatibility complex-related genes up-regulated in MSI-H colorectal cancer (P < 0.05, Benjamini and Hochberg False Discovery Rate)**	

**Gene name**	**Fold change**

Major histocompatibility complex, class II, DR alpha	2.0
Major histocompatibility complex, class II, DQ beta 1	1.8
Major histocompatibility complex, class II, DP alpha 1	1.8
H. sapiens HLA-DMA gene	1.7
Major histocompatibility complex, class II, DR beta 1	1.6
Major histocompatibility complex, class II, DR beta 5	1.5
HLA-B associated transcript 1	1.2
Major histocompatibility complex, class I, C	1.2

It is of interest that reduction of the stringency of our statistical comparison to P < 0.05 (Benjamini and Hochberg False Discovery Rate) generates a list of 4788 differentially expressed genes (Additional Table 1 ). These include several more genes with key immunomodulatory functions. Transcripts specific to co-stimulatory molecules (CD80 and CD86), HSP60, MHC peptides, pro-inflammatory cytokine receptors, Perforin 1 and Caspase 9 were amongst those that were up-regulated in MSI-H colorectal cancers. These subsidiary data provide further evidence that these cancers excite an antigen-specific immune response. Closer study of HLA molecules that were up-regulated in MSI-H cancers reveals that the majority are Class II-related (Table 3). This finding is consistent with previous studies [24,25] and supports the notion that immunogenicity of these cancers relies on antigen presentation by Antigen Presenting Cells (cross-priming) rather than directly by the tumour. The HLA Class I molecule β^2^-microglobulin has been shown to be a target for mutation in the MSI-H pathway and this renders HLA Class I machinery ineffective in these tumours [26]. This gene does not, however, appear in our list of differentially expressed genes.

This study compares gene expression profiles in 133 primary human cancers and confirms the previous finding on a smaller sample set that microarray profiling can differentiate cancers according to microsatellite stability status [27]. A recent report on differential gene expression from microarray profiling of smaller numbers of MSI-H (n = 8) and MSS (n = 14) colorectal cancer tissue samples yielded findings similar to ours in genes such as TP53, IGF2, RAN, MORF4L1, ZFP36L2 and CCNF [28]. Their observation that EIF3S2 is downregulated in MSI-H was confirmed in our study, as was the downregulation of TGFβ RII. However, they did not observe differences in MMR genes, such as hMLH1 and PMS2, or indeed the immunomodulatory genes that we report. The likely explanation is that the smaller sample numbers used in their analysis as well as the smaller size of their spotted cDNA array (8000 genes) limits the sensitivity of their microarray comparisons. Previously two groups have reported the results of cDNA microarray comparisons of MSI-H and MSS cancer cell lines but both were restricted to very small numbers [29,30]. Interestingly, of the 122 differentially expressed genes identified by these two studies 33 transcripts (27%) were also included in our list of 4788 genes (P < 0.05, Benjamini and Hochberg False Discovery Rate). However, some genes noted to be down-regulated in MSI-H cancer cell lines were up-regulated in the MSI-H cancers in our analysis, and vice versa. These disparities can be attributed to the fact that cancer cells cultured *in vitro *behave differently to cells from primary tumours. Indeed Bertucci *et al *report that colorectal cancer lines show overexpression of genes involved in cellular proliferation and underexpression of several gene clusters, including a cluster associated with immunomodulatory genes, when compared to colorectal cancer tissue samples using microarrays [28]. These biological differences and the small numbers used in the cell line experiments render any concordance between our data and cell line analyses altogether encouraging.

We acknowledge that mRNA profiles cannot be presumed to reflect functional significance at a protein level. However, the upregulation of such a large number of genes, known to be involved in innate and antigen-specific immune responses, in MSI-H colorectal cancer indicates a genuine difference in host-tumour interactions. These findings are entirely novel and add considerable weight to the argument that MSI-H cancers excite an immune response. Clearly, additional work on protein expression specific to the genes we have identified will help to elucidate the exact nature of immune mechanisms in these cancers.

In this study microdissection was deliberately eschewed as our focus was the interaction between tumour, stroma and inflammatory cells and the expression profiles therefore reflect contributions from each of these groups. Microdissection would have excluded the contribution of certain cell populations which may have important roles in the modulation of host-tumour interactions. We have previously extensively analysed the composition of tumour biopsies obtained by sampling exophytic areas of resected specimens. We consistently found that a random sample taken from a fresh frozen segment of tumour tissue contained at least 80% tumour cells (Unpublished data). We know from experience that necrotic tumour does not yield high quality RNA and our study included only those samples that yielded high quality RNA and thus, the sampling technique we used inherently excludes necrotic tumour. It is therefore unlikely that our results are attributable to the presence of tumour necrosis. Furthermore, a DNA microarray analysis of the gene expression profiles of naïve versus activated tumour-specific lymphocytes did not show differences of gene expression in heat shock proteins or the interleukins noted in our study [31]. This suggests that our observations do not simply reflect the more pronounced lymphocyte infiltrates of MSI-H colorectal cancer although we accept that this remains a possibility.

## Conclusions

Certainly, our results provide new evidence to support the notion that MSI-H colorectal cancers are immunogenic and new insights into the pathways that may be involved. Further focussed study of these cancers may clarify the immunology of colorectal cancer and, specifically, may provide useful targets for directing immunotherapeutic strategies.

## Methods

### Patient population and microsatellite analysis

The Local Ethics Committee approved this study and all patients gave informed consent prior to surgery. Tissue was available from a bank of 223 colorectal cancers resected at The Royal London Hospital between December 1997 and March 2003. Tissue samples of tumour and normal mucosa were taken within 20 minutes of resection and snap frozen in liquid nitrogen. Normal mucosa and tumour DNA was extracted and used in PCR reactions to amplify the mononucleotide BAT-26 marker, as described elsewhere [32]. Products were separated and visualised on micro-fabricated chips to identify tumours displaying bandshifts characteristic of a high degree of microsatellite instability [33].

### Microarray profiling and analysis

Total RNA was prepared from samples using an RNeasy^® ^kit (QIAGEN, Hilden, Germany) and quality was assessed on the Agilent Bioanalyser 2100. Only 129 samples, from our tumour bank, that yielded high quality mRNA with minimal degradation and clear 18S/28S ribosomal peaks, were included in the analysis. Preparation of *in vitro *transcription (IVT) products, oligonucleotide array hybridization and scanning were performed according to Affymetrix^® ^(Santa Clara, California) protocols. In brief, 5 μg of total RNA from each colon tumour and T7-linked oligo-dT primers were used for first-strand cDNA synthesis. IVT reactions were performed in batches to generate biotinylated cRNA targets, which were chemically fragmented at 95°C for 35 minutes. Fragmented biotinylated cRNA (10 μg) was hybridized at 45°C for 16 hours to Affymetrix^® ^high density oligonucleotide array human HG-U133A chip, which contains 22,283 probe sets representing more than 14,500 well-substantiated human genes. The arrays were washed and stained with streptavidin-phycoerythrin (SAPE, final concentration of 10 μg/ml). Signal amplification was performed using a biotinylated anti-streptavidin antibody. The array was scanned according to the manufacturer's instructions (Affymetrix Genechip^® ^Technical Manual, 2001). Scanned images were inspected for the presence of obvious defects (artefacts or scratches) on the array. Defective chips were excluded and the sample was re-analysed. To minimize discrepancies due to variables such as sample preparation, hybridization conditions, staining, or array lot, the raw expression data was scaled using Affymetrix^® ^Microarray Suite 5.0 software. The trimmed mean signal of all probe sets on the HG-U133A chip was adjusted to a user-specified target signal value (1500) for each array for global scaling. No specific exclusion criteria were applied.

Comparative analysis between expression profiles for MSI-H and MSS samples was carried out on GeneSpring™ software version 6.0 (Silicongenetics, Redwood, California). The "Cross gene error model for deviation from 1.0" was active. Gene expression data was normalised in two ways: "per chip normalisation" and "per gene normalisation". For "per chip normalisation" all expression data on a chip is normalised to the 50^th ^percentile of all values on that chip. For "per gene normalisation" the data for a given gene is normalised to the median expression level of that gene across all samples. The data sets are then assigned to the two groups MSI-H and MSS, and the expression profiles of the two groups were compared using unpaired t-tests (with Welch's correction for unequal variances) and multiple testing corrections to identify genes that were differentially expressed between them.

### RT-PCR Analysis

Nine genes were further analysed using quantitative real-time RT-PCR. To compare similar groups each MSI-H tumour was matched to an MSS tumour from patients of similar age, the same side of the colon and, where possible, same Dukes' Stage. Sample RNA was extracted, quantified and quality-controlled as for microarray analysis. High-quality RNA was available on 28 tumours of the 29 tumours with MSI-H. These were matched to a group of 26 MSS cancers (Table 1). Three analyses were performed on smaller groups (Table 4) because high-quality RNA was no longer available for analysis. Gene-specific primers and probe sets for each gene (Table 4) were obtained from Assays-on-Demand Gene Expression Products (Applied Biosystems, Warrington, UK). RT-PCR reactions were carried out in a one-tube system for seven genes but reactions for two genes were sub-optimal in this set-up and so a two-tube system was used instead (Table 4). Sample RNA was processed in duplicate with serial dilutions of Human Universal Reference RNA (Stratagene, LaJolla, California), in triplicate, and No Template Controls on the same 96-well plate. Standard curves were constructed from the Universal RNA wells with arbitrary units of 1 Unit equivalent to 1 picogram of Universal RNA. Duplicate wells that differed by more that one Ct count were repeated or were excluded from the analysis. The results were compared using the non-parametric Mann-Whitney test and significance was taken at P < 0.05.

**Table 4:** Summary of RT-PCR details and the numbers of samples in each analysis. Analysis of hMLH1, IL-15 and Caspase2 restricted to smaller numbers due to limited availability of high-quality RNA.

Gene name	GENBANK NUMBER	Chemistry (Applied Biosystems, Warrington, UK)	Sample Numbers (*n*)	
			MSS	MSI-H

hMLH1	NM_000249	High-Capacity cDNA Archive Kit + TaqMan Universal PCR Master Mix (with AmpErase UNG)	12	16
TP53	NM_000546	TaqMan^® ^EZ-RT-PCR Kit	26	27
HSP-70 kD 1B	NM_005346	TaqMan^® ^EZ-RT-PCR Kit	26	28
HSP-110	NM_014278	TaqMan^® ^EZ-RT-PCR Kit	26	28
IL-18	NM_001562	TaqMan^® ^EZ-RT-PCR Kit	24	28
IL-8	NM_000584	TaqMan^® ^EZ-RT-PCR Kit	26	28
IL-15	NM_000585	High-Capacity cDNA Archive Kit + TaqMan Universal PCR Master Mix (with AmpErase UNG)	12	16
Granulysin	NM_006433	TaqMan^® ^EZ-RT-PCR Kit	26	27
Caspase 2	NM_001224	TaqMan^® ^EZ-RT-PCR Kit	21	18

## List of abbreviations

MSI = Microsatellite instability

MSI-H = High degree microsatellite instability

MSS = Microsatellite stable (including low degree instability)

## Authors' contributions

AB carried out RT-PCR experiments and analysis, microarray data analysis and drafted the manuscript.

SA participated in microarray experiments and carried out microarray data analysis.

RH, FH, XH and PS coordinated and performed microarray experiments.

RF performed histopathological analysis of cancers.

SB and SD conceived the study, supervised the project and drafted the manuscript.

All authors read and approved the final manuscript.

## Supplementary Material

Additional File 1 2070 genes differentially expressed between MSI-H and MSS colorectal cancers (P < 0.005, Benjamini and Hochberg False Discovery Rate). 4788 genes differentially expressed between MSI-H and MSS colorectal cancers (P < 0.05, Benjamini and Hochberg False Discovery Rate). 542 genes differentially expressed between MSI-H and MSS colorectal cancers (P < 0.05, Bonferoni Mutilple Testing Correction). Click here for file

Additional File 2 2184 genes differentially expressed between MSI-H (HNPCC profiles excluded) and MSS colorectal cancers (P < 0.005, Benjamini and Hochberg False Discovery Rate). 1436 genes upregulated in sporadic MSI-H cancers (with fold change). 748 genes upregulated in MSS cancers (with fold change). 4874 genes differentially expressed between MSI-H and MSS colorectal cancers (P < 0.05, Benjamini and Hochberg False Discovery Rate). Click here for file
